# Anti-melanogenic effects of hydroxyethyl chrysin through the inhibition of tyrosinase activity: *In vitro* and *in silico* approaches

**DOI:** 10.1016/j.heliyon.2025.e41718

**Published:** 2025-01-04

**Authors:** Yuna Lee, Ha-Yeon Song, Eui-Baek Byun

**Affiliations:** Advanced Radiation Technology Institute, Korea Atomic Energy Research Institute, Jeongeup, 56212, Republic of Korea

**Keywords:** *Melanogenesis*, *B16F10 cells*, *Molecular docking*

## Abstract

The suppression of tyrosinase (TYR), a key enzyme in melanogenesis, has been suggested as an effective strategy for preventing melanin accumulation. We previously discovered the novel chrysin derivative hydroxyethyl chrysin (HE-chrysin) through an irradiation technique, which exerted higher anti-inflammatory and anti-cancer activities than original chrysin. In the present study, we explored whether HE-chrysin has antioxidant and anti-melanogenic capacity using *in vitro* B16F10 murine melanoma cells and *in silico* molecular docking. HE-chrysin exhibited enhanced antioxidant capacity in DPPH, ABTS, and FRAP assays, and it decreased cellular H_2_O_2_-stimulated reactive oxygen species levels in comparison to original chrysin. At 2.5 μM, HE-chrysin reduced 3-isobutyl-1-methylxanthine-stimulated melanin production significantly by suppressing intracellular TYR activity without cytotoxicity. Furthermore, molecular docking showed that HE-chrysin inhibited TYR activity by interacting with key residues (Glu^256^ and Asn^260^) and chelating Cu^+^ ions at the active site, with a binding free energy of −7.00 kcal/mol compared with arbutin (−5.12 kcal/mol). Our findings show that HE-chrysin is an anti-melanogenic candidate and a potential antioxidant for use in dermatologic therapy.

## Graphical abstract

**Figure:**
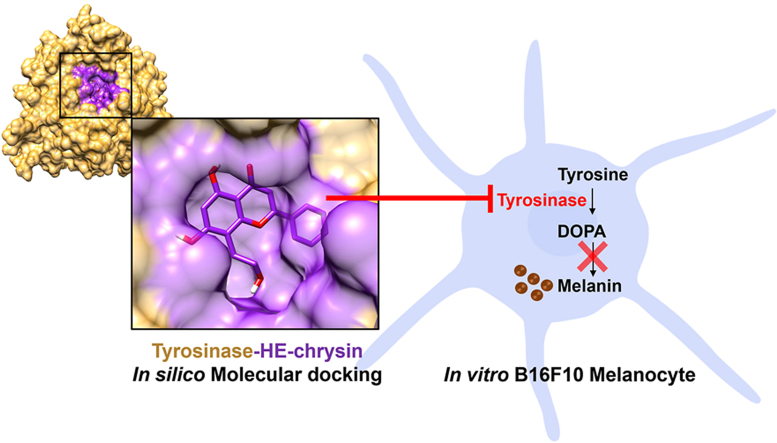

## 1 Introduction

Hyperpigmentation is a common dermatologic disorder that causes darkening or discoloration of the skin, which affected persons may find bothersome. Melanin is produced in epidermal melanocytes and plays an essential role in skin color [1]. It also shields the skin against ultraviolet (UV) radiation; however, accumulation and over-synthesis of melanin can result in hyperpigmentation [2]. The various forms of hyperpigmentation include lentigines, post-inflammatory pigmentary alterations, ephelides, and melasma. Such conditions are associated with psychosocial distress and adversely affect both quality of life and self-perception [3]. Melanogenesis is modulated by tyrosinase (TYR), tyrosinase-related protein 1 (TRP-1), and tyrosinase-related protein 2 (TRP-2). TYR is a critical regulator in melanin synthesis since it catalyzes the rate-limiting reaction of melanogenesis [4]. Furthermore, TYR facilitates the conversion of tyrosine to 3,4-dihydroxy phenylalanine (DOPA) and DOPA to DOPA-quinone, which is then oxidized by TRP-2 to produce 5,6-dihydroxyindole-2-carboxylic acid (DHICA). TRP-1 then catalyzes the oxidation of DHICA, resulting in the production of brown-black eumelanin [5]. In the presence of cysteine, DOPA-quinone reacts to form pheomelanin, a yellow-red pigment. Melanogenesis is also closely linked to the generation of reactive oxygen species (ROS), which are produced in melanocytes stimulated by UV radiation. ROS accumulation can trigger the aging of the skin and result in melanocyte proliferation and hyperpigmentation [6]. Furthermore, ROS can induce melanin synthesis by upregulating protein expression of TYR [7]. Thus, TYR inhibitors and antioxidants with ROS scavenging activities could be used to inhibit melanogenesis in the epidermis. Various anti-TYR agents, including arbutin [8], kojic acid [9], and ellagic acid [10], have been utilized as skin-whitening agents; however, they possess undesirable characteristics and may elicit side effects linked to instability, carcinogenicity, and low bioavailability [4]. Arbutin acts by competitively binding to the active site of tyrosinase, thereby inhibiting its enzymatic activity; however, its glucosylated structure can be hydrolyzed by metabolic enzymes to release hydroquinone, raising safety concerns due to potential cytotoxicity [11]. Kojic acid inhibits tyrosinase by directly chelating Cu^+^ ions at the active site, but prolonged use has been associated with phototoxicity and possible carcinogenicity [12]. Additionally, prolonged use of these agents may lead to skin irritation or sensitization [13,14]. As a result, there has been increasing interest in identifying novel TYR inhibitors derived from both synthetic and natural substances to address such limitations and improve their application in skincare products. Excessive ROS production due to UV exposure is one of the main risk elements of melanogenesis; therefore, polyphenolic compounds with both anti-TYR and antioxidant properties have been proposed as promising candidates for development of safer and more effective anti-melanogenesis treatments [15].

**Fig. 1:**
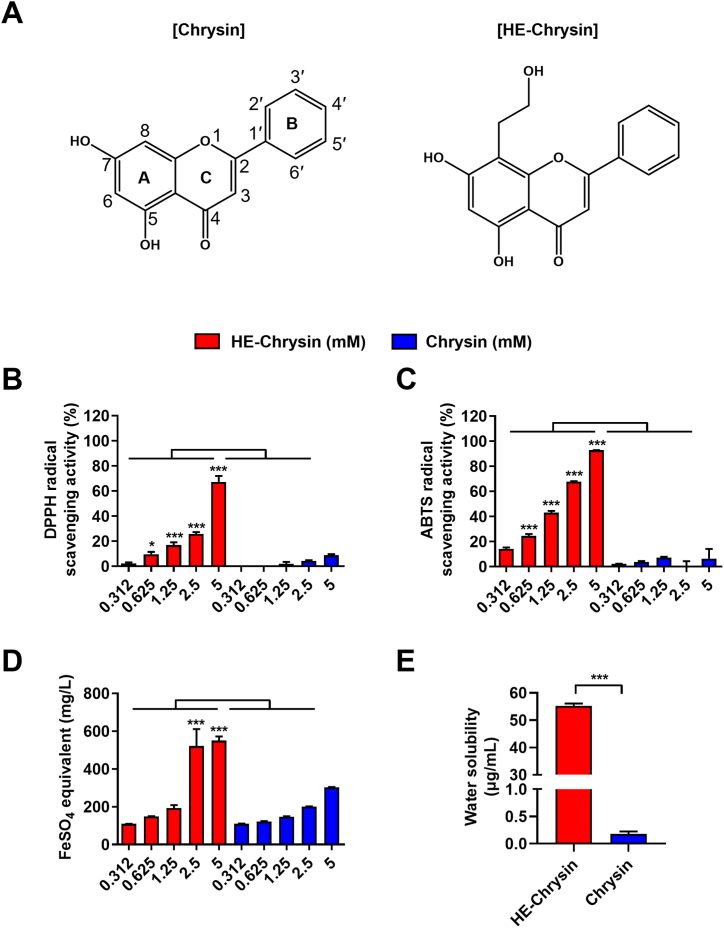
HE-Chrysin exhibited higher antioxidant capacity and water solubility than original chrysin. (A) Chemical structure of chrysin (left) and HE-chrysin (right). Various doses of chrysin and HE-chrysin (0.312–5 mM) were allowed to react with DPPH (B), ABTS (C), and FRAP (D) radical solution and subjected to antioxidant assays. Results are presented as mean ± SD (*n* = 3); ∗*p* < 0.05, ∗∗∗*p* < 0.001. Differences were tested using one-way ANOVA with Tukey's *post-hoc* test. (E) The water-solubility of chrysin and HE-chrysin was determined. Results are presented as mean ± SD (*n* = 3). Differences were tested using unpaired, two-tailed Student's *t*-test. ∗∗∗*p* < 0.001.

Many polyphenolic compounds possess diverse biological activities, including dual roles as anti-TYR and antioxidant, along with anti-inflammatory properties. These multifunctional attributes make them promising candidates for next-generation skincare products that prioritize safety and efficacy [16]. Among these, 5,7-Dihydroxyflavone (chrysin), one of the most abundant polyphenolic compounds in honey and propolis, has been the subject of extensive research for many years due to its diverse physiological benefits, including antioxidant [17], anti-inflammatory [18], and anti-cancer activities [19]. Previous studies have indicated that chrysin may exert anti-photoaging and anti-melanogenesis effects in human dermal fibroblasts and B16 murine melanoma cells by blocking TYR activity and attenuating the expression of melanogenic proteins, involving TYR, TRP-1, and TRP-2 [20]. Moreover, *in silico* docking studies have suggested that chrysin interacts with the active site of TYR, which has a binding free energy of −5.3 kcal/mol [21]. However, since chrysin is insoluble in water, it has extremely poor bioavailability (<1 %) and efficacy in humans, which limits its potential medicinal applications [22]. Consequently, medicinal applications of chrysin have been explored based on diverse derivatives produced through chemical synthesis or biotransformation [[23], [24], [25]]. In previous studies, we showed that the chrysin derivative hydroxyethyl chrysin (HE-chrysin; Fig. 1A), generated by γ-irradiation from chrysin methanolic solution, exerted stronger anti-cancer [26] and anti-inflammatory [27] effects than original chrysin. Given its multifunctional roles, we hypothesized that HE-chrysin could serve as a novel TYR inhibitor, addressing the limitations of conventional agents such as arbutin. These attributes suggest HE-chrysin's potential as a safer and more effective alternative for future skincare formulations. To verify this hypothesis, the present study aimed to examine the physiological activities of HE-chrysin to specifically explore its potential antioxidant and anti-melanogenic effects. We investigated whether HE-chrysin can eliminate ROS production and inhibit TYR activity essential for melanin synthesis using *in vitro* B16F10 murine melanoma cells. Moreover, we conducted *in silico* molecular docking analyses to explore the binding interactions between HE-chrysin and TYR and to elucidate the structure-activity relationships.

## 2 Material and methods

### 2.1 Chemicals

2,2′-Azino-bis(3-ethyl-benzthiazoline-6-sulfonic acid) (ABTS), arbutin, chrysin, 2′,7′-dichlorodihydrofluorescein diacetate (DCFH-DA), dimethyl sulfoxide (DMSO), 2,2-diphenyl-1-picrylhydrazyl (DPPH), 3-isobutyl-1-methylxanthine (IBMX), DOPA, 3-(4,5-dimethylthiazol-2-yl)-2, 5-diphenyl tetrazolium bromide (MTT), phenylmethylsulfonyl fluoride (PMSF), 2,4,6-tripyridyl-s-triazine (TPTZ), and Triton X-100 were acquired from Sigma-Aldrich (St. Louis, MO, USA). Anti-TYR (sc-20035), anti-TRP-1 (sc-514,911), and anti-TRP-2 (sc-271,356) antibodies were acquired from Santa Cruz Biotechnology (Dallas, TX, USA). Anti-α-tubulin (2144 S) was obtained from Cell Signaling Technology (Danvers, MA, USA). Horseradish peroxidase (HRP)-conjugated anti-mouse immunoglobulin G (IgG) antibody was obtained from Calbiochem (#401215, San Diego, CA, USA), and HRP-conjugated anti-rabbit IgG antibody was obtained from Abcam (ab6721, Cambridge, MA, USA).

### 2.2 Preparation of HE-chrysin

HE-chrysin, a chrysin derivative produced by γ-irradiation, was prepared as described previously by Song et al. (2015), with minor modifications [26]. A solution of chrysin in methanol (2 mM) was gamma-irradiated at 10 kGy for 5 h using a cobalt-60 irradiator (AECL, IR-79, MDS Nordion Inc., Ottawa, Canada). Subsequent to irradiation, HE-chrysin was purified using a preparative high-performance liquid chromatography (HPLC) system (1260 series, Agilent Technologies, Santa Clara, CA, USA) equipped with a Zorbax Eclipse XDB-C18 column (21.2 × 150 mm inner diameter, 7 μm particle size) and a diode array detector (DAD; 280 nm). A gradient solvent system comprising solvent A (0.1 % formic acid in water) and B (100 % methanol) was initiated with 20 % methanol and then increased to 40, 55, and 80 % at 12, 25, and 30–38 min, respectively. The flow rate was 1 mL/min. The HE-chrysin detected at 19.5 min was isolated by the HPLC-DAD (Fig. S1), and the isolated HE-chrysin (purity of >95 %) was evaporated and freeze dried to obtain powdered samples. The recovery yield, calculated as the ratio of the initial amount of chrysin to the final amount of purified HE-chrysin, was approximately 15 % (data not shown).

### 2.3 Antioxidant capacity

DPPH, ABTS, and ferric reducing antioxidant power (FRAP) assays were employed to determine the antioxidant capacity of chrysin and HE-chrysin (at 0.312, 0.625, 1.25, 2.5, and 5 μM) [[28], [29], [30]]. In the DPPH assay, 0.1 mL of a sample was combined with 0.1 mL of 0.2 mM DPPH methanolic solution. After incubation for 15 min in the dark, absorbance was recorded at 517 nm using a SpectraMax M3 multi-detect microplate reader (Molecular Devices, Sunnyvale, CA, USA). For the ABTS assay, a working solution was prepared by combining 7 mM ABTS stock solution (5 mL) with 2.45 mM potassium persulfate (0.088 mL), followed by 16 h incubation in the dark at room temperature (RT). The ABTS radical solution was adjusted with distilled water to an absorbance of 1.2 (±0.2) at 750 nm before usage. Subsequently, 0.1 mL of each sample was mixed with 0.1 mL of ABTS working solution. After 30 min of incubation in the dark, absorbance was recorded at 760 nm. The percentage of DPPH or ABTS radical scavenging activity was calculated according to the following equation:

$$\%\;\text{Radical}\;\text{scavenging}\;\text{activity}=\left(\frac{\text{Absorbance}\;\text{Control}‐\text{Absorbance}\;\text{Sample}}{\text{Absorbance}\;\text{Control}}\right)\times 100$$

For the FRAP assay, a working reagent was produced by combining 300 mM acetate buffer (pH 3.6), 10 mM TPTZ solution (dissolved in 40 mM HCl), and 20 mM ferric chloride (10:1:1). Subsequently, 50 μL of sample was mixed with 750 μL of FRAP reagent. Following 15-min incubation, absorbance was recorded at 593 nm. The FRAP values were determined using the calibration curve of FeSO_4_ (Sigma-Aldrich) and presented as FeSO_4_ equivalent (mg/L).

### 2.4 Water solubility assay

Water solubility is crucial for assessing the bioavailability and potential applications of dermatological products in aqueous environments, including biological fluids or hydrophilic skin layers. Water solubility experiments were conducted as described previously, with slight changes [31]. Chrysin (10 mg) and HE-chrysin (10 mg) samples were dissolved in 10 mL of distilled water and sonicated using a TECAN ultra-sonicator (Osaka, Japan) for 1 h to prepare saturated solutions. The samples were then centrifuged at 2000×*g* for 10 min, and the supernatants underwent filtering through 0.45-μm membrane filters to eliminate undissolved chemicals. The absorbances of each of the chrysin- and HE-chrysin aqueous solutions were measured using an HPLC-UV-DAD (1260 series, Agilent Technologies) equipped with a Zorbax Eclipse XDB-C18 column. A gradient solvent system comprising solvent A (0.1 % formic acid in water) and B (100 % methanol) was initiated with 20 % methanol and then increased to 40, 55, and 80 % at 12, 25, and 30–38 min, respectively. The flow rate was 1 mL/min. The concentration of each saturated aqueous solution was determined using the standard curves derived from corresponding methanol solutions (ranging from 0 to 100 μg/mL; Fig. S2) at wavelengths of 268 and 294 nm for chrysin and HE-chrysin, respectively.

### 2.5 Cell culture and cell viability assay

B16F10 murine melanoma cells (cell line CRL-6475) were obtained from the American Type Culture Collection (Manassas, VA, USA). The B16F10 cells were grown in high-glucose Dulbecco's modified Eagle's medium (DMEM; WelGene, Daegu, Korea) containing 10 % fetal bovine serum (Gibco BRL, Grand Island, NY, USA) and 100 U/mL penicillin/streptomycin (Gibco BRL) at 37 °C in a humidified atmosphere with 5 % CO_2_. Cell viability was assessed using MTT and annexin V/propidium iodide (PI) assays, using a previously reported method with minor modifications [32]. For the MTT assay, B16F10 cells were plated in a 96-well plate (5 × 10^3^ cells/well) for 4 h and then treated with chrysin or HE-chrysin in 0.1 % DMSO/DMEM to obtain final concentrations (2.5, 5, and 10 μM). Following 72-h incubation at 37 °C with 5 % CO_2_, the media were removed, and the cells were treated with 50 μL of MTT solution (0.5 mg/mL in DMEM) for 2 h. Thereafter, the supernatant was eliminated, and the produced formazan crystals were dissolved in 0.1 mL of DMSO. A microplate reader was employed to determine the absorbance values of sample solutions at 570 nm.

For the annexin V/PI assay, B16F10 cells were seeded in a 6-well plate (3 × 10^4^ cells/well). After 4-h incubation, chrysin or HE-chrysin dissolved in 0.1 %DMSO/DMEM were added to each well to achieve final concentrations of 1.25 and 2.5 μM, respectively. Following 72-h incubation, the cells were collected using 0.25 % trypsin-ethylenediaminetetraacetic acid solution (Gibco BRL) and washed two times with phosphate-buffered saline (PBS). Afterward, the collected cells were employed to the assay with an annexin V/PI detection kit (#556547, BD Biosciences, San Jose, CA, USA) according to the manufacturer's guidelines. Apoptotic cells were quantified using a flow cytometer (MACSQuant VYB, Miltenyi Biotec, Bergisch Gladbach, Germany), and the data were analyzed using FlowJo (ver. 10; TreeStar, Ashland, OR, USA).

Normal human primary epidermal keratinocytes (NHEK; PCS-200-011, ATCC) were cultured in 154CF medium (Gibco BRL) supplemented with 1 × Human Keratinocyte Growth Supplement (Gibco BRL), 0.07 mM CaCl_2_ (Gibco BRL), and 100 U/mL penicillin/streptomycin, at 37 °C with 5 % CO_2_. A WST-1 assay was performed to assess cell cytotoxicity [33] using an EZ-Cytox cell viability assay kit (DAEIL Lab, Seoul, Korea). NHEK cells were seeded in a 96-well plate (3 × 10^3^ cells/well) for 24 h and treated with chrysin or HE-chrysin dissolved in 0.1 % DMSO/154CF medium (1.25, 2.5, and 5 μM). The NHEK cells treated with 0.1 % DMSO in 154CF medium were used as a control. A blank control consisting of 10 μL of the kit solution and 90 μL of 154CF medium without cells was also included. Following 48-h incubation, 10 μL of the kit solution was added to each well and further incubated for 4 h. Cell viability was determined by measuring the produced formazan at 450 nm using a microplate reader. Cell viability was expressed as a relative value compared to the control.

### 2.6 Measurement of ROS production

Intracellular ROS levels were measured using an oxidant-sensing probe, DCFH-DA, based on a previously described method with minor modifications [32]. B16F10 cells were cultured in a 6-well plate (5 × 10^4^ cells/well) and pre-loaded with 10 μM DCFH-DA for 30 min in the dark at 37 °C. Subsequently, the cells were rinsed two times using serum-free DMEM and treated with chrysin (2.5 μM), HE-chrysin (2.5 μM), or Trolox (2 mM; positive control) for 1 h. Thereafter, the cells were simulated with 500 μM H_2_O_2_ for 30 min. The cells were harvested, and the fluorescence signals of dichlorofluorescein (DCF) were analyzed using flow cytometry.

### 2.7 Measurement of melanin content and TYR activity

IBMX is a widely used pharmacological inducer of melanogenesis; it accelerates TYR activity by elevating intracellular cyclic adenosine monophosphate (cAMP) levels [34]. UV-B radiation induces melanin production indirectly, and α-melanocyte-stimulating hormone activates the cAMP pathway through binding to melanocortin 1 receptor; however, IBMX directly increases cAMP levels by inhibiting phosphodiesterase, providing a controlled and efficient method for consistent melanin production without the risks of DNA damage or hormonal side effects [35,36]. Therefore, the intracellular melanin content and TYR activity were assessed using IBMX stimulation, according to previously described methods, with slight modifications [32,37]. B16F10 cells were cultured in a 6-well plate (3 × 10^4^ cells/well) for 4 h. The cells were then treated with chrysin, HE-chrysin, or arbutin (positive control) in the presence of IBMX stimulation for 72 h. The final concentrations of the mixtures were as follows: chrysin, 1.25 and 2.5 μM; HE-chrysin, 1.25 and 2.5 μM; arbutin, 500 μg/mL; and IBMX, 50 μM. Cells treated with 0.1 % DMSO in DMEM were used as the control. After incubation, the cells were lysed in 1 M NaOH containing 10 % DMSO at 80 °C for 1 h. The melanin content was then measured at 405 nm using a microplate reader and calculated using a calibration curve of synthetic melanin (0–500 g/mL; Sigma-Aldrich).

To assess intracellular TYR activity, cells were seeded in a 6-well plate (3 × 10^4^ cells/well). After 4-h incubation, the cells were treated with chrysin, HE-chrysin, or arbutin in the presence of IBMX stimulation for 72 h. Their final concentrations were as described above. Afterward, the cells were lysed in 1 % Triton X-100 in 100 mM phosphate buffer (pH 6.8) containing 1 mM PMSF (to prevent proteolytic degradation during cell lysis), followed by centrifugation (16,000 × *g*, 15 min, 4 °C) to obtain the cell lysates as a supernatant. Samples were maintained on ice throughout the procedure to preserve enzyme activity and prevent degradation. Subsequently, the supernatant (90 μL) was transferred to a 96-well plate, and 5 mM DOPA dissolved in 100 mM phosphate buffer (10 μL) was added to each well. A blank control, consisting of lysis buffer (90 μL) and DOPA solution (10 μL) was included to account for background absorbance. After 1-h reaction at 37 °C, absorbance at 475 nm was measured and the dopachrome content in each well was expressed as a relative value compared with that in the control.

### 2.8 Investigation of intracellular protein levels

The intracellular levels of TYR, TRP-1, and TRP-2 in B16F10 cells were investigated by western blotting and flow cytometry, using previously described methods with minor modifications [26,32]. For western blotting, cells were lysed in radioimmunoprecipitation assay buffer (#89900, Thermo Fisher Scientific, Waltham, MA, USA) containing protease inhibitor cocktail (#78425, Thermo Fisher Scientific), maintained on ice for 15 min, and then centrifuged (16,000 × *g*, 15 min, 4 °C) to obtain the cell lysates as a supernatant. The lysates (30 μg protein) were separated by a 10 % sodium dodecyl sulfate-polyacrylamide gel electrophoresis and subsequently transferred onto a polyvinylidene difluoride membrane (#1620177, Bio-Rad, Hercules, CA, USA). After blocking with General-Block solution (TransLab, Daejeon, Korea) for 1 h, the membrane was reacted overnight at 4 °C with primary antibodies, i.e., anti-TYR (1:100 dilution), anti-TRP-1 (1:400 dilution), anti-TRP-2 (1:200 dilution), or α-tubulin (1:1000 dilution) antibodies. Then, the membrane was reacted with HRP-conjugated anti-mouse (1:5000 dilution) or anti-rabbit (1:5000 dilution) secondary antibodies for 2 h at RT. Bands of target proteins were visualized with ECL-plus detection reagents (#1863096, #1863097, Thermo Fisher Scientific) using a ChemiDoc Imaging System (Bio-Rad). For flow cytometry, cells were harvested and incubated with 2 % bovine serum albumin dissolved in PBS for 15 min. The cells were subsequently fixed and permeabilized using a Cytofix/Cytoperm kit (#555028, BD Biosciences) according to the manufacturer's guidelines. The cells were then stained with anti-TYR, anti-TRP-1, and anti-TRP-2 for 30 min at 4 °C. After washing two times, the cells were reacted for 30 min with secondary antibodies (goat anti-rabbit Alexa Fluor 568 or goat anti-mouse Alexa Flour 488; 1:500 dilution). The intracellular levels of TYR, TRP-1, and TRP-2 in B16F10 cells were detected using flow cytometry.

### 2.9 Preparation of ligands and protein structures

The chemical structures of chrysin, HE-chrysin, and arbutin were reconstructed using ChemDraw (ver. 19.1), and their stable conformations were determined through MM2 calculations in Chem3D (ver. 19.1). The crystal structure of TYR protein (2Y9X) [15], with a high resolution of 2.3 Å, was acquired from the RCSB Protein Data Bank (https://www.rcsb.org). This structure, derived from mushroom *Agaricus bisporus*, is used extensively in computational studies [38,39] due to its detailed representation of the active site, including conserved Cu^+^ ions and catalytically essential residues. The structure was changed to eliminate unrequired parts using PyMOL ver. 2.3.4. Out of the eight-chain structures of TYR, only chain A, with 391 amino acids and two Cu^+^ ions, Cu^400^ and Cu^401^, was preserved for the docking study. Energy minimization of the resulting protein was performed using Swiss-PDB Viewer (ver. 4.1).

### 2.10 Molecular docking

Molecular docking of arbutin, chrysin, and HE-chrysin against TYR was conducted using AutoDock Tools (ver. 1.5.6) [40]. Briefly, polar hydrogen atoms and Kollman charges were incorporated into the TYR structure. Gasteiger charges were applied to the ligands, and non-polar hydrogen atoms were combined. For the docking analysis, the ligand-protein binding region was enclosed in a box with the number of grid points in *x* × *y* × *z* directions (40 × 40 × 40) and a grid spacing of 0.375 Å. The grid-box center was defined as *x* = −9.985, *y* = −28.542, and *z* = −43.490 to encompass the active region of TYR [41], which is constituted of six residues, i.e., His^61^, His^85^, His^94^, His^259^, His^263^, and His^296^ (Fig. S3). A Lamarckian genetic algorithm was employed to conduct computational calculations, with the search parameters set to 50 runs. Default values were applied to the other docking parameters. At the end of docking, the best conformation with the lowest binding energy (kcal/mol) was selected. Two and three-dimensional interactions of the ligands at the active site of TYR were visualized using PyMOL and LigPlot^+^ (ver. 2.2.8), respectively.

### 2.11 In silico ADMET estimations

The pharmacokinetics profiles of HE-chrysin, including adsorption, distribution, metabolism, excretion, and toxicity (ADMET), were estimated using the admetSAR online server (https://lmmd.ecust.edu.cn/admetsar1) [42].

### 2.12 Statistical analyses

All experiments were replicated at least thrice, yielding uniform data. Data are presented as means ± standard deviation (SD). Differences were tested using one-way analysis of variance (ANOVA) with Tukey's *post-hoc* test or an unpaired two-tailed Student's *t*-test. Statistical significance is reported at ∗*p* < 0.05. All statistical analyses were conducted using GraphPad Prism (ver. 8; San Diego, CA, USA).

## 3 Results and discussion

### 3.1 Effect of HE-chrysin on antioxidant ability and water solubility

The antioxidant potential of HE-chrysin (0.312–5 mM) was assessed using DPPH, ABTS, and FRAP activity analyses. Antioxidant activity refers to the ability to scavenge free radicals, ROS, and nitrogen species through the donation of hydrogen atoms (protons) or electrons [43]. The DPPH and ABTS assays measure free radical neutralization via hydrogen atom and electron transfer, and the FRAP assay evaluates reducing power as electron-donating capacity. These assays comprehensively assess the antioxidant potential of HE-chrysin through diverse mechanisms and oxidative species. The results were compared with those obtained for the original chrysin. In the DPPH assay (Fig. 1B), HE-chrysin showed a radical scavenging activity of 10 % at 0.625 mM, 17 % at 1.25 mM, 25 % at 2.5 mM, and 67 % at 5 mM, while chrysin exhibited a lower activity across all concentrations, with less than 10 % at 5 mM. Similarly, in the ABTS assay (Fig. 1C), HE-chrysin demonstrated a radical scavenging activity of 24 % at 0.625 mM, 43 % at 1.25 mM, 68 % at 2.5 mM, and 93 % at 5 mM, showing much greater efficacy than chrysin, whose radical scavenging activity remained below 10 % across all concentration. In the FRAP assay (Fig. 1D), HE-chrysin also exhibited a significantly higher ferric-reducing potential than chrysin, with FeSO_4_ equivalents of 522 mg/L at 2.5 mM and 550 mg/L at 5 mM, whereas those of chrysin were less than 300 mg/L at all concentrations. These results indicate that HE-chrysin is markedly more effective than chrysin in scavenging free radicals and reducing oxidative species.

HE-chrysin (55 ± 0.78 μg/mL) was also 50-fold more water-soluble than chrysin (0.18 ± 0.05 μg/mL; Fig. 1E). This enhanced water solubility is likely due to hydroxyethylation, which increases the polarity of the molecule and allows for stronger hydrogen bonding with water molecules. Owing to the formation of H-bonds between water and hydroxyl groups in molecules, molecules with more hydroxyl groups exhibit higher polarity and water-solubility [44]. As a result, this increased water solubility enhances skin penetration and distribution, making HE-chrysin a promising candidate for topical medicinal applications.

The superior antioxidant activity and water solubility of HE-chrysin can be attributed to its structural modification, specifically to the introduction of a hydroxyethyl group at the C8 position. This modification likely enhances the electro-donating capacity of HE-chrysin, contributing to its higher radical scavenging ability. The hydroxyethyl group has been reported to enhance various functional properties in small molecules. For example, Singh et al. (2016) demonstrated that incorporating a hydroxyethyl group in the piperazine moiety significantly boosted radical scavenging activity [45]. Similarly, Lin et al. (2017) showed that hydroxyethylation of flavone derivatives improved antibacterial activity, possibly because of enhanced solubility and stronger interactions with bacterial membranes [46]. Additionally, hydroxyethylated rutin (troxerutin) exhibits substantially improved water solubility and bioavailability, thereby expanding its range of bioactivity compared with unmodified rutin, which is limited by poor bioavailability [47]. Overall, the findings demonstrate that hydroxyethylation improves the antioxidant potential and solubility of chrysin significantly, highlighting HE-chrysin as a promising candidate for pharmaceutical and cosmetic applications.

### 3.2 Scavenging effect of HE-chrysin on H_2_O_2_-stimulated ROS generation in B16F10 cells

**Fig. 2:**
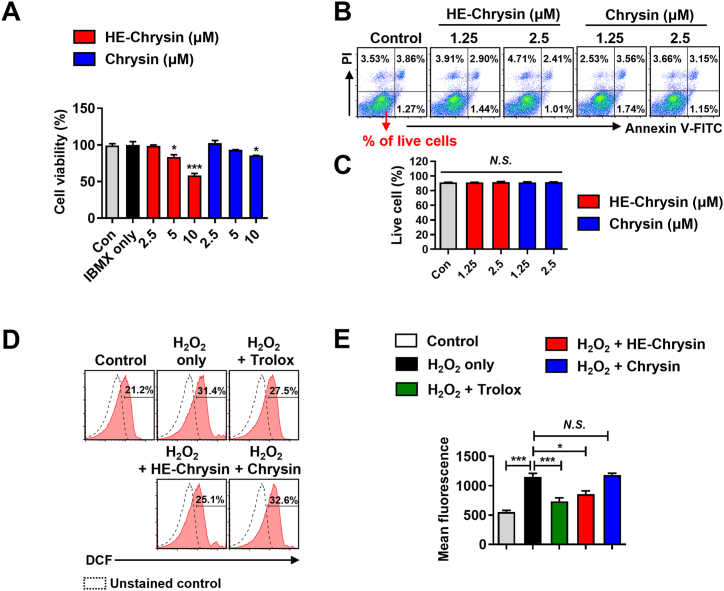
HE-chrysin inhibited H_2_O_2_-induced ROS production in comparison to original chrysin in B16F10 cells. The cells were treated with chrysin (2.5, 5, and 10 μM), HE-chrysin (2.5, 5, and 10 μM), or IBMX (50 μM). After 72-h incubation, cell viability was determined via the MTT assay (A) and the annexin V/PI assay (B and C). (B) Representative dot plot image showing the annexin V/PI assay. (C) Live cells were gated on annexin V^−^PI^-^ cells; *N.S.*, not significant at *p* < 0.05. (D and E) Cells were pre-loaded with DCFH-DA, incubated with chrysin or HE-chrysin (2.5 μM) for 1 h, and then treated with H_2_O_2_ (0.5 mM) for 30 min. (D) Representative histogram showing the percentage of DCF (ROS level). (E) Bar graph illustrating the mean fluorescence of DCF. The data are mean ± SD (*n* = 3). Differences were tested using one-way ANOVA with Tukey's *post-hoc* test. ∗*p* < 0.05, ∗∗∗*p* < 0.001; *N.S.*, not significant at *p* < 0.05.

To assess whether the enhanced antioxidant capacity of HE-chrysin was maintained at the intracellular level, we examined its ROS scavenging activity under H_2_O_2_-stimulated oxidative stress in B16F10 murine melanoma cells. Before the experiment, cell viability was assessed using the MTT and annexin V/PI assays to select non-cytotoxic doses of chrysin and HE-chrysin. The MTT assay was used to measure mitochondrial activity, providing an indirect assessment of cell viability, and the annexin V/PI assay was used to directly detect early apoptosis and necrosis, offering complementary insights into cellular health [48]. The findings of both tests matched, indicating no cytotoxicity at concentrations ≤2.5 μM for both chrysin and HE-chrysin (Fig. 2A–C). Consequently, the optimal concentration of chrysin and HE-chrysin was set at 2.5 μM for the ROS experiment. We investigated the ROS scavenging activity of chrysin (2.5 μM) and HE-chrysin (2.5 μM) in B16F10 cells using the fluorescence probe DCFH-DA (Fig. 2D and E). The DCF fluorescence intensity of B16F10 cells exposed to H_2_O_2_ indicated increased ROS generation (1150 ± 84, *p* < 0.001) when compared with that in the control group (553 ± 40). In contrast, treatment with Trolox (positive control; 732 ± 86, *p* < 0.001) or HE-chrysin (856 ± 80, *p* < 0.05) before H_2_O_2_ treatment decreased the generation of intracellular ROS levels considerably, whereas treatment with chrysin did not. This observation indicates that HE-chrysin can effectively suppress ROS production stimulated by H_2_O_2_ in B16F10 cells.

### 3.3 Melanogenesis inhibitory effect of HE-chrysin in B16F10 cells

**Fig. 3:**
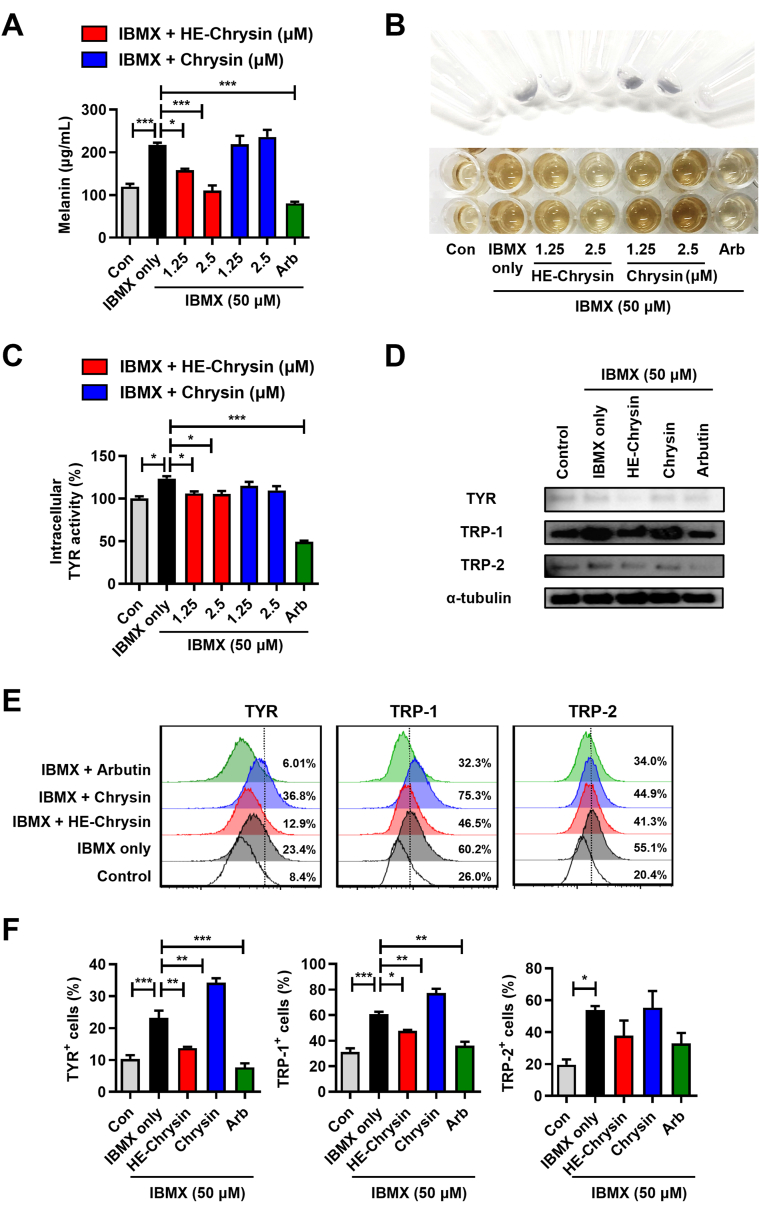
HE-chrysin suppressed melanin synthesis, intracellular TYR activity, and TYR-related proteins in IBMX-stimulated B16F10 cells. The cells were treated with chrysin or HE-chrysin (1.25 and 2.5 μM) in the presence of IBMX (50 μM) for 72 h. Arbutin (500 μg/mL) was used as a positive control. (A and B) Effect of chrysin and HE-chrysin on IBMX-induced melanin production. The melanin content was measured using an NaOH dissolution assay. (B) Representative image of melanin contents before (above image) and after (below image) cell dissolution. (C) Effect of chrysin and HE-chrysin on IBMX-induced TYR activity. Intracellular TYR activity was assessed via a DOPA oxidation assay. Data are displayed as the mean ± SD (*n* = 3). Differences were assessed using one-way ANOVA with Tukey's *post-hoc* test; ∗*p* < 0.05, ∗∗∗*p* < 0.001. (D–F) Melanogenesis-related protein expression in IBMX-stimulated B16F10 cells. The protein levels of TYR, TRP-1, and TRP-2 were detected by Western blotting and flow cytometry analyses. (D) α-Tubulin was used as the control protein for western blotting. (E) Representative histogram showing the percentages of TYR (Alexa fluor 568), TRP-1 (Alexa fluor 488), and TRP-2 (Alexa fluor 488). (F) Bar graph illustrating the mean ± SD of TYR^+^, TRP-1^+^, or TRP-2^+^ cells (*n* = 3). Differences were tested using one-way ANOVA with Tukey's *post-hoc* test; ∗*p* < 0.05, ∗∗*p* < 0.01, ∗∗∗*p* < 0.001.

IBMX increases melanin generation and controls the melanogenesis pathway [49]. Therefore, we examined the suppression effects of HE-chrysin on melanogenesis in IBMX-stimulated B16F10 cells. The cells were incubated with chrysin (1.25 and 2.5 μM), HE-chrysin (1.25 and 2.5 μM), or arbutin (positive control; 500 μg/mL), in the presence of IBMX. Arbutin was used as a positive control at a non-cytotoxic concentration of 500 μg/mL (Fig. S4). The IBMX-stimulated melanin content of B16F10 cells treated with HE-chrysin (1.25 μM, 158 ± 5 μg/mL, *p* < 0.05; 2.5 μM, 111 ± 20 μg/mL, *p* < 0.001) or arbutin (80 ± 6.8 μg/mL, *p* < 0.001) was significantly lower than that of the group treated with IBMX only (218 ± 9 μg/mL), whereas no significant effect of chrysin treatment was observed (Fig. 3A and B). Similarly, the groups treated with HE-chrysin (1.25 μM, 106 ± 4 %, *p* < 0.05; 2.5 μM, 105 ± 5 %, *p* < 0.05) or arbutin (49 ± 2 %, *p* < 0.001) effectively inhibited intracellular TYR activity compared with the IBMX only group (Fig. 3C). Moreover, the expression of melanogenic enzymes, including TYR, TRP-1, and TRP-2, was attenuated through treatment with HE-chrysin or arbutin in IBMX-stimulated B16F10 cells (Fig. 3D). Flow cytometry results indicated that the intracellular levels of TYR (*p* < 0.01) and TRP-1 (*p* < 0.05) decreased considerably in the cells treated with HE-chrysin or arbutin compared with those in IBMX-stimulated B16F10 cells (Fig. 3E and F). TRP-2 was also decreased upon HE-chrysin treatment in IBMX-stimulated B16F10 cells; however, the difference was not significant.

**Fig. 4:**
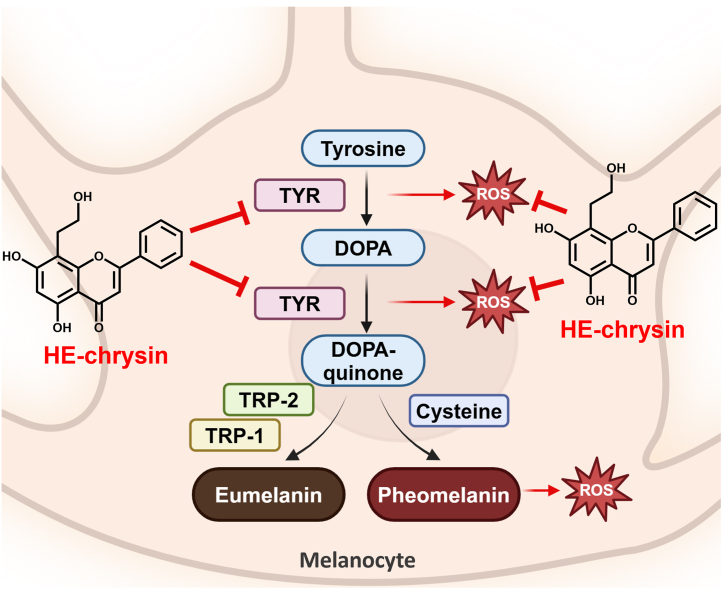
Role of HE-chrysin in melanogenesis and oxidative stress regulation.

During melanogenesis, tyrosine is oxidized to DOPA and DOPA-quinone by tyrosinase, a process generating ROS [4,5]. DOPA-quinone is a critical branch point, directing the synthesis of either eumelanin or pheomelanin depending on cysteine availability. While eumelanin exhibits photoprotective properties, pheomelanin is associated with increased ROS production and oxidative stress. Our findings indicate that HE-chrysin effectively scavenges ROS and inhibits tyrosinase activity, thereby reducing DOPA-quinone formation, modulating melanogenesis, and mitigating oxidative damage (Fig. 4). Moreover, the absence of cytotoxicity in human epidermal keratinocytes at concentrations of up to 5 μM (Fig. S5) underscores the potential of HE-chrysin as a safe and effective ingredient for skin-whitening agents in the pharmaceutical or cosmetic industries.

### 3.4 Molecular docking analysis of HE-chrysin to TYR

*In silico* molecular docking analysis of arbutin (positive control), chrysin, and HE-chrysin against the TYR enzyme was performed to elucidate their inhibitory effects on TYR activity. The active center of TYR involves two Cu^+^ ions, Cu^400^ and Cu^401^, which are chelated by six amino acids, including His^61^, His^85^, His^94^, His^259^, His^263^, and His^296^. Cu^400^ reportedly interacts with His^61^, His^85^, and His^94^, whereas Cu^401^ interacts with His^259^, His^263^, and His^296^ [41]. The imidazole ring of histidine has a strong affinity for metal ions such as Cu^2+^, enabling the stabilization of the active site [50]. These Cu^+^ ions play a critical role in the enzymatic oxidation of tyrosine by facilitating electron transfer during its conversion to DOPA and subsequently DOPA-quinone (Fig. 4). Disrupting the coordination of Cu^+^ ions with the active site residues through chelation impairs electron transfer, suppressing TYR enzymatic activity [41]. In addition, other active residues, including His^244^, Glu^256^, Asn^260^, Phe^264^, Met^280^, and Val^283^, form a hydrophobic cavity that is essential for tyrosinase catalytic activity [51].

**Fig. 5:**
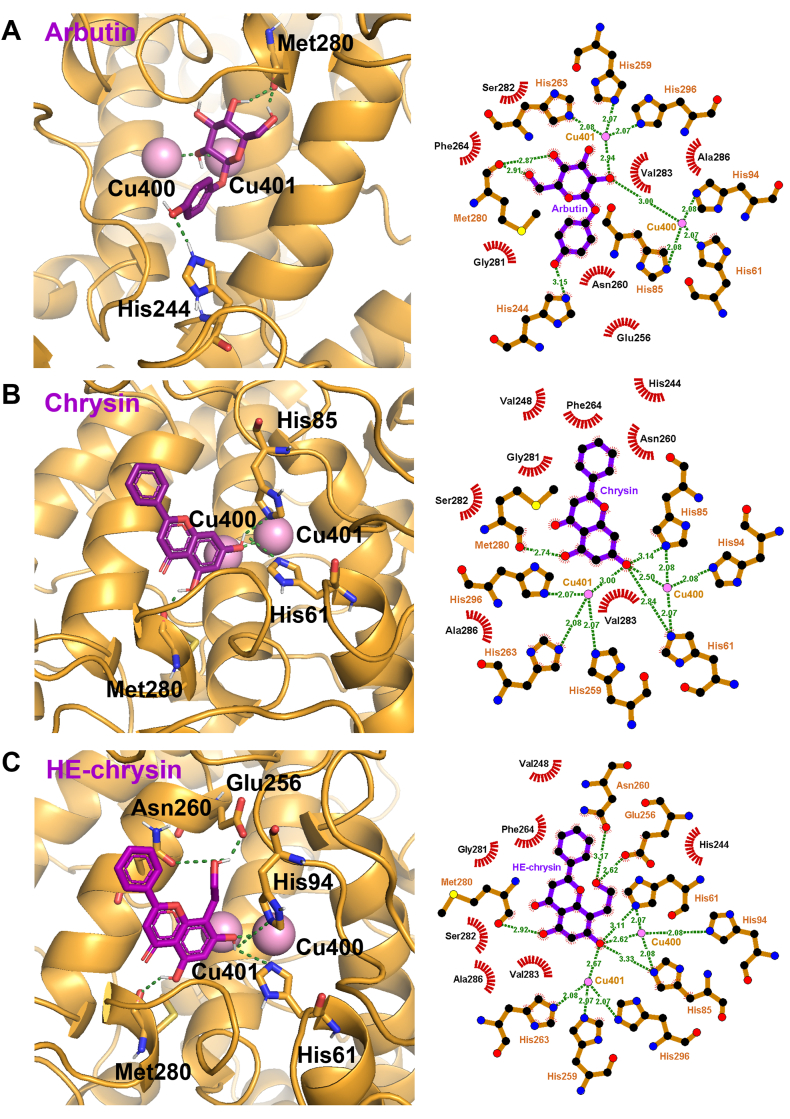
HE-chrysin had high binding affinity with TYR. Molecular docking poses of (A) arbutin, (B) chrysin, and (C) HE-chrysin at the active region of TYR. The 3D interactions between the docked ligands and the active center of TYR were visualized using PyMOL. The docked 2D views were generated using LigPlot^+^. H-bonds are presented by green dashed lines with distances, while hydrophobic interactions are indicated by semi-circular red spokes. The colors that correspond to the elements are as follows: C atom (black), H atom (white), N atom (blue), O atom (red), and copper ions (pink).

The binding free energies (ΔG_bind_) and binding interactions of arbutin, chrysin, and HE-chrysin against TYR are summarized in Table 1. The ΔG_bind_ of arbutin with TYR was found to be −5.12 kcal/mol. Arbutin was observed to form H-bonds with His^244^ and Met^280^, and to have hydrophobic interactions with Glu^256^, Asn^260^, Phe^264^, Gly^281^, Ser^282^, Val^283^, and Ala^286^ (Fig. 5A). These findings are consistent with those reported by Lee et al. (2019), who observed that arbutin formed H-bonds with His^244^ and Met^280^ and interacted with Asn^260^, Phe^264^, Ser^282^, Val^283^, and Ala^286^ of TYR [52]. In addition, the oxygen atom of arbutin has been observed to chelate both Cu^+^ ions (Cu^400^ and Cu^401^), as noted by Thongchai et al. (2009) [53].

**Table 1:** Summary of binding interactions of the TYR active site with ligands.

Ligands	Δ*G*_bind_ (kcal/mol)	Hydrogen-bond interacting residues	Hydrophobic bond interacting residues
Arbutin	−5.12	His^244^, Met^280^, Cu^400^, Cu^401^	Glu^256^, Asn^260^, Phe^264^, Gly^281^, Ser^282^, Val^283^, Ala^286^
Chrysin	−6.39	His^61^, His^85^, Met^280^, Cu^400^, Cu^401^	His^244^, Val^248^, Asn^260^, Phe^264^, Gly^281^, Ser^282^, Val^283^, Ala^286^
HE-chrysin	−7.00	His^61^, His^85^, Glu^256^, Asn^260^, Met^280^, Cu^400^, Cu^401^	His^244^, Phe^264^, Gly^281^, Ser^282^, Val^283^, Ala^286^

The ΔG_bind_ of chrysin against TYR was calculated to be −6.39 kcal/mol. At the active region of TYR, chrysin formed H-bonds with His^61^, His^85^, and Met^280^ and hydrophobic bonds with His^244^, Val^248^, Asn^260^, Phe^264^, Gly^281^, Ser^282^, Val^283^, and Ala^286^ (Fig. 5B). In addition, the Cu^+^ ions were chelated by the oxygen atom of chrysin. The ΔG_bind_ of HE-chrysin against TYR was calculated to be −7.00 kcal/mol, which was lower than that of chrysin (−6.39 kcal/mol), indicating favorable binding interactions between HE-chrysin and TYR. This was consistent with the observed intracellular TYR inhibitory effect (Fig. 3C). Furthermore, HE-chrysin formed H-bonds with His^61^, His^85^, Glu^256^, Asn^260^, and Met^280^, and it interacted hydrophobically with His^244^, Phe^264^, Gly^281^, Ser^282^, Val^283^, and Ala^286^ (Fig. 5C). The oxygen atom of HE-chrysin was observed to interact with both Cu^400^ and Cu^401^, indicating that the suppression effect of HE-chrysin on TYR was achieved by chelation with Cu^+^ ions, similar to arbutin. In addition, through interactions with the aforementioned residues, HE-chrysin may closely interact with the active region of TYR. Notably, the H-bonds established between the hydroxyl group derived from hydroxyethylation and the active region of TYR (Glu^256^ and Asn^260^) likely elicit the greater anti-TYR effect of HE-chrysin than the original chrysin in the *in vitro* analysis. This observation is consistent with the report of Si et al. (2013) [54], indicating that Glu^256^ and Asn^260^ of TYR, together with His^85^, are key interacting residues leading to the inhibitory effect of TYR. Xue et al. (2022) also reported that Asn^260^ as well as His^85^, His^244^, and His^259^ are the key interacting residues involved in the inhibition of TYR activity [55]. The results demonstrate that H-bonding and hydrophobic interactions between HE-chrysin (or arbutin) and TYR play substantial roles in inhibiting enzymatic activity.

### 3.5 Predicted ADMET profile of HE-chrysin

The ADMET predictions for HE-chrysin suggest favorable pharmacokinetic properties (Table S1). HE-chrysin was predicted to cross the blood-brain barrier and exhibit high human intestinal absorption, indicating good bioavailability. It was classified as a non-substrate and non-inhibitor for key CYP450 enzymes, implying a low risk of drug-drug interactions. Additionally, HE-chrysin was predicted to be non-mutagenic and non-carcinogenic, highlighting its safety. However, further *in vivo* studies are needed to substantiate these predictions and comprehensively confirm the pharmacokinetic and safety profiles of HE-chrysin.

## 4 Conclusion

Our investigations using *in vitro* B16F10 cell experiments and *in silico* molecular docking analyses suggest that HE-chrysin has significant potential as an antioxidant and anti-melanogenic agent. The observed suppression effect of HE-chrysin on melanogenesis was attributed to its ability to suppress intracellular TYR activity, which is essential for melanin synthesis. The high binding affinity of HE-chrysin toward TYR (ΔG_bind_, −7.00 kcal/mol) was consistent with the suppression effect of HE-chrysin on TYR activity in the *in vitro* analysis. The inhibition of TYR activity by HE-chrysin was achieved by interaction with key residues of TYR, namely Glu^256^ and Asn^260^, and by chelation with the Cu^+^ ions of the TYR enzyme. To further validate the efficacy of HE-chrysin, future studies will need to include animal and human experiments. Our results demonstrate that the chrysin derivative, HE-chrysin, is a promising candidate for novel skin-whitening agents in the pharmaceutical or cosmetic industries.

## CRediT authorship contribution statement

**Yuna Lee:** Writing – original draft, Visualization, Methodology, Formal analysis. **Ha-Yeon Song:** Writing – original draft, Visualization, Methodology, Funding acquisition, Formal analysis. **Eui-Baek Byun:** Supervision, Funding acquisition, Conceptualization.

## Data availability

Data will be made available on request.

## Declaration of competing interest

The authors declare that they have no known competing financial interests or personal relationships that could have appeared to influence the work reported in this paper.
