# Supplementary material for: Anti-melanogenic effects of hydroxyethyl chrysin through the inhibition of tyrosinase activity: In vitro and in silico approaches
Source: Heliyon. 2025 Jan 4;11(2):e41718. doi: 10.1016/j.heliyon.2025.e41718 (PMC11774776; doi:10.1016/j.heliyon.2025.e41718)
Supplement: Multimedia component 1 [file mmc1.docx]

**Anti-melanogenic effects of hydroxyethyl chrysin through the inhibition of tyrosinase activity: *in vitro* and *in silico* approaches**

Yuna Lee^a†^, Ha-Yeon Song^a†^, and Eui-Baek Byun^a*^

^a^Advanced Radiation Technology Institute, Korea Atomic Energy Research Institute, Jeongeup 56212, Republic of Korea

^†^These authors contributed equally to this work.

^*^**Corresponding author**: Eui-Baek Byun, Advanced Radiation Technology Institute, Korea Atomic Energy Research Institute, Jeongeup 56212, Republic of Korea

Tel.: +82-63-570-3245, Fax: +82-63-570-3371

E-mail: [ebbyun80@kaeri.re.kr](mailto:ebbyun80@kaeri.re.kr) (E-B Byun)

**
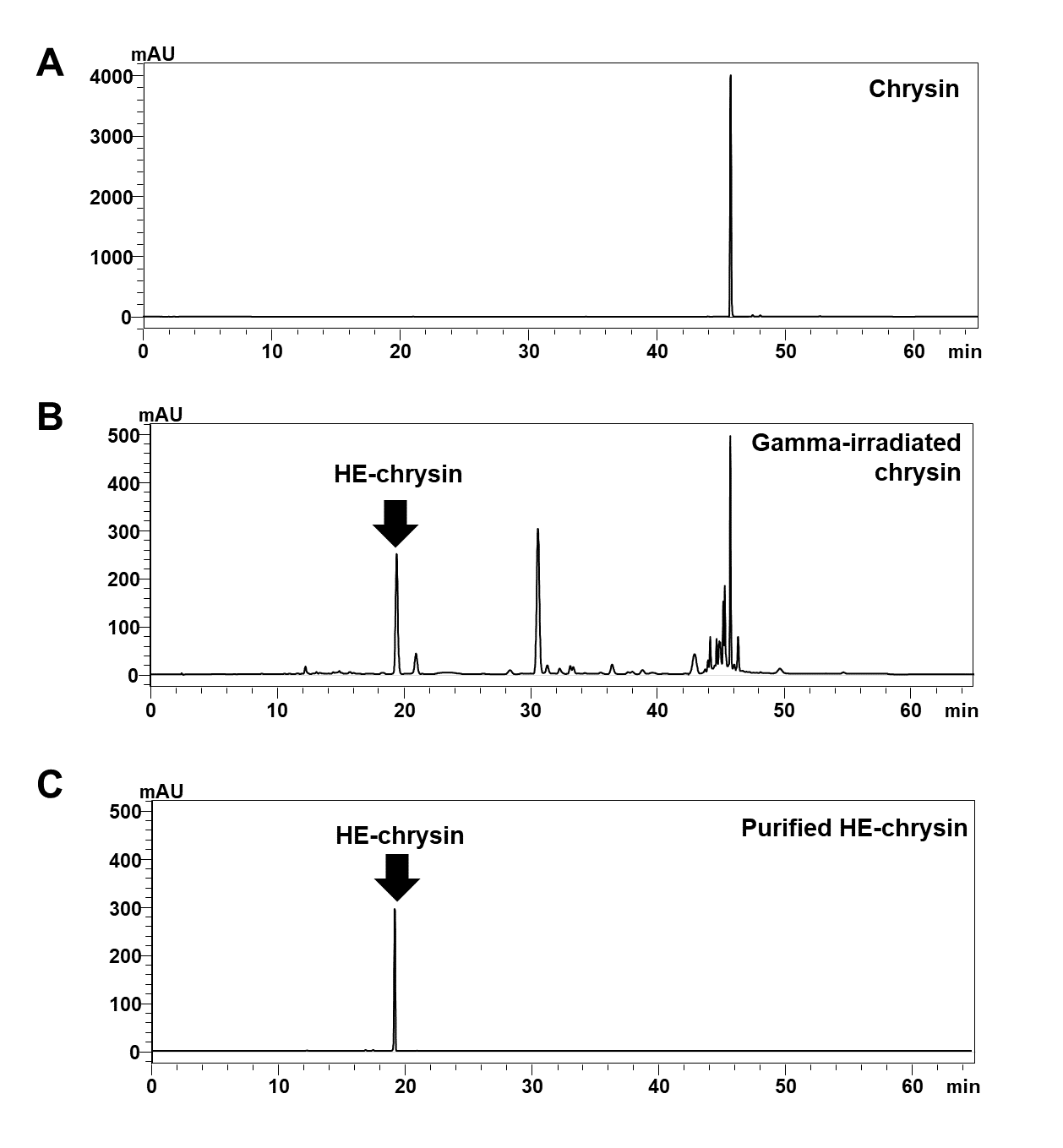
**

**Fig. S1.** HPLC-DAD chromatograms of (A) chrysin, (B) gamma-irradiated chrysin (10 kGy for 5 h), and (C) purified HE-chrysin.

**
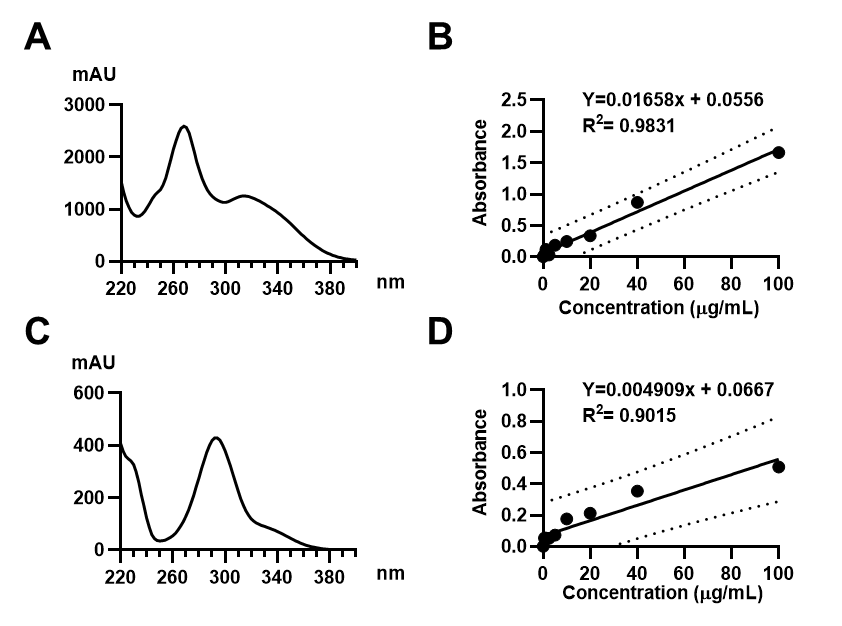
**

**Fig. S2.** UV spectrum and standard curve of chrysin and HE-chrysin for water solubility determination. UV scanning spectra (A) and standard curve of chrysin methanolic solution (B). The chrysin standard curve was determined at 268 nm. UV scanning spectra (C) and standard curve of HE-chrysin mathanolic solution (D). The calibration curve of HE-chrysin was determined at 294 nm.


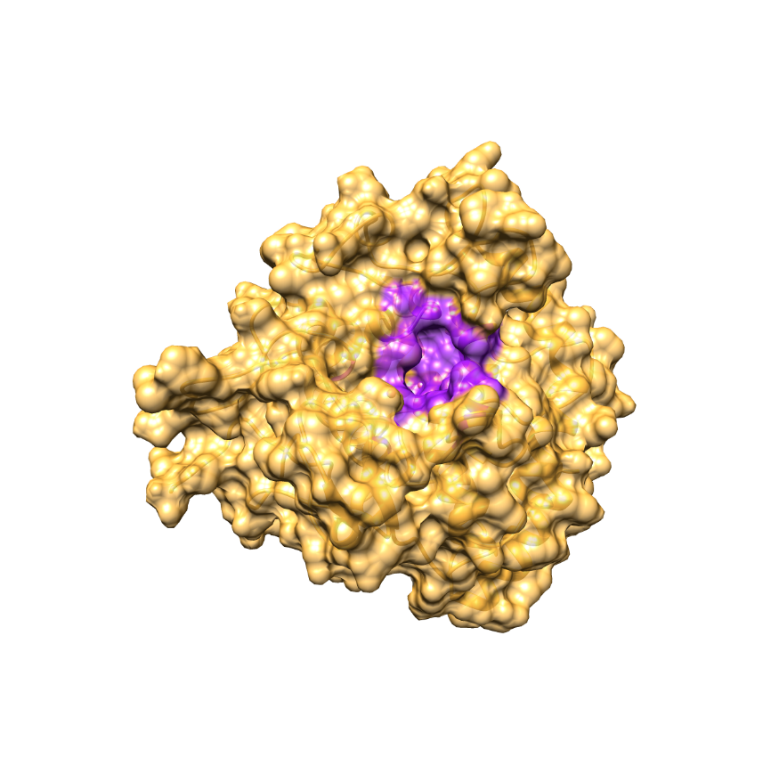


**Fig. S3.** TYR active binding site highlighted in purple.

**
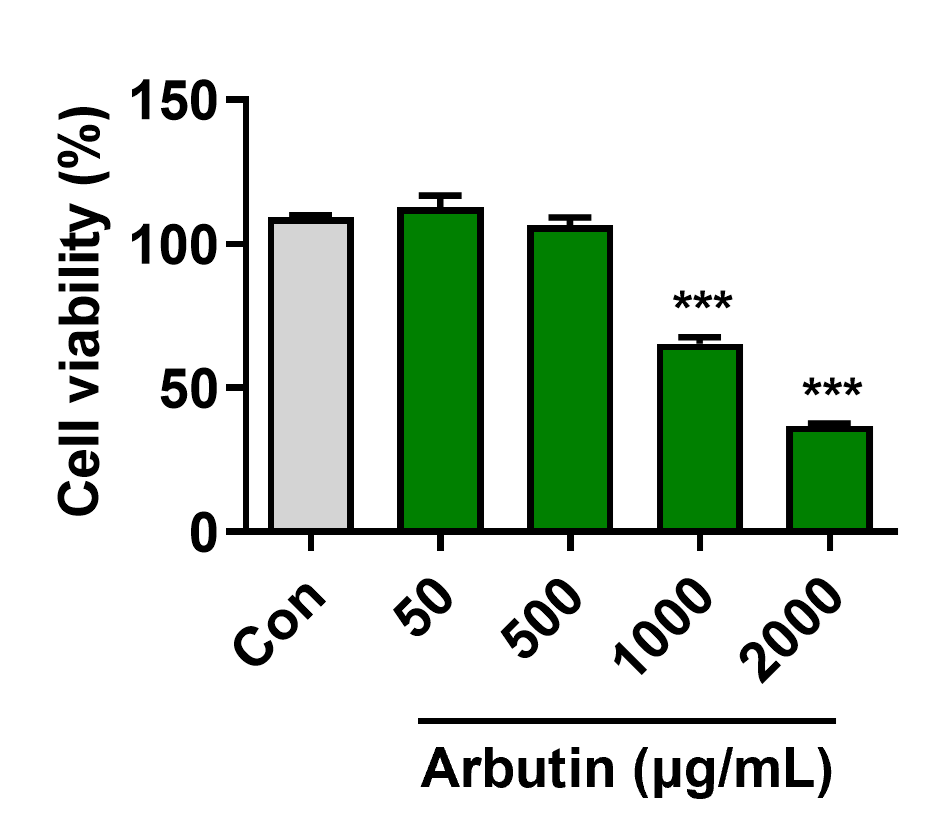
**

**Fig. S4.** Impact of HE-chrysin on cell viability in B16F10 cells. The cells were treated with arbutin (50, 500, 1000, and 2000 µg/mL). After 72-h incubation, cell viability was determined via the MTT assay. The data are the mean ± SD (*n* = 3). Differences were tested using one-way ANOVA with Tukey’s *post-hoc* test. ^***^*p* < 0.001


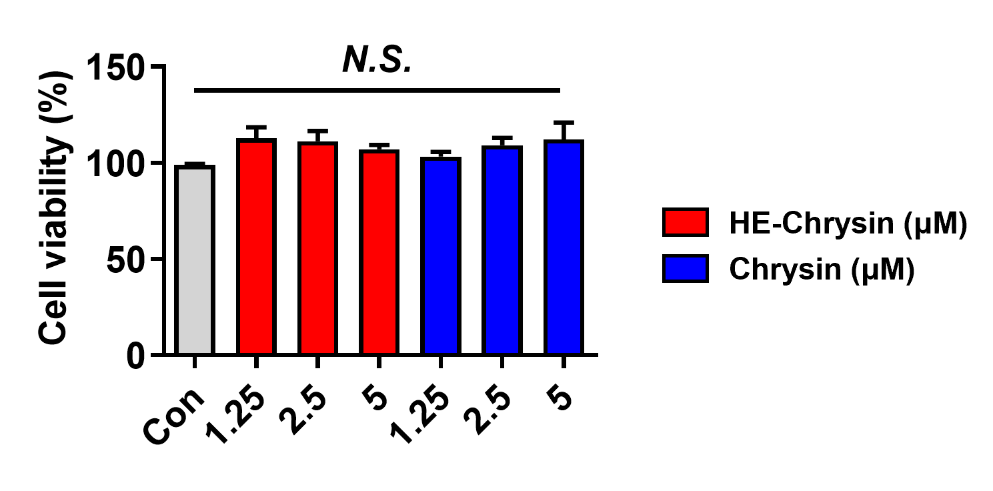


**Fig. S5.** Impact of HE-chrysin on cell viability in NHEK cells. The cells were treated with chrysin (1.25, 2.5, and 5 µM) or HE-chrysin (1.25, 2.5, and 5 µM). After 48-h incubation, cell viability was determined by the WST-1 assay. The data are the mean ± SD (*n* = 3). Differences were tested using one-way ANOVA with Tukey’s *post-hoc* test. *N.S.*, not significant at *p* < 0.05.

**Table S1.** *In silico* ADMET predictions of HE-chrysin

| **Absorption** | |
| --- | --- |
| Blood-brain barrier | BBB^+^ |
| Human intestinal absorption | HIA^+^ |
| **Metabolism** | |
| CYP450 2C9 substrate | Non-substrate |
| CYP450 2D6 substrate | Non-substrate |
| CYP450 3A4 substrate | Non-substrate |
| CYP450 1A2 inhibitor | Non-inhibitor |
| CYP450 2C9 inhibitor | Non-inhibitor |
| CYP450 2D6 inhibitor | Non-inhibitor |
| CYP450 2C19 inhibitor | Non-inhibitor |
| CYP450 3A4 inhibitor | Non-inhibitor |
| **Toxicity** | |
| AMES Toxicity | Non-inhibitor |
| Carcinogens | Non-carcinogens |
